# Water deficit‐induced oxidative stress affects artemisinin content and expression of proline metabolic genes in *Artemisia annua* L.

**DOI:** 10.1002/2211-5463.12184

**Published:** 2017-01-25

**Authors:** Priyanka Soni, Malik Z. Abdin

**Affiliations:** ^1^ Department of BiotechnologyCentre for Transgenic Plant DevelopmentJamia Hamdard UniversityNew DelhiIndia

**Keywords:** *Artemisia annua*, ornithine δ‐aminotransferase, pyrroline‐5‐carboxylase dehydrogenase, Δ1‐pyrroline‐5‐carboxylase reductase, Δ1‐pyrroline‐5‐carboxylate synthetase

## Abstract

Water stress is one of the most critical abiotic stresses that restricts growth, development, and alters physiological and biochemical mechanisms of plant. The effects of long‐term water shortage‐induced oxidative stress on morphophysiological parameters, proline metabolic genes, and artemisinin content were studied in *Artemisia annua* L. under greenhouse conditions. Plant growth, biomass accumulation, relative water content, and chlorophyll content were reduced under drought. Leaf water potential ranged from −0.3248 MPa to −1.22 MPa in stress conditions. Increased levels of proline accumulation, protein concentration, and lipid peroxidation were detected in water‐stressed plants. Stage‐dependent increases in activity of antioxidants including superoxide dismutase, ascorbate peroxidase, glutathione reductase, monodehydroascorbate reductase, and dehydroascorbate reductase were observed. The expression of proline biosynthetic genes including pyrroline‐5‐carboxylase synthase1, 1‐pyrroline‐5‐carboxylase synthase2, and 1‐pyrroline‐5‐carboxylase reductase was induced, while the ornithine aminotransferase transcript showed a variable response and the expression of proline catabolic genes including proline dehydrogenase1, proline dehydrogenase1, and proline 5‐carboxylate dehydrogenase was reduced by water stress. Our results indicate that the glutamine pathway is predominant under drought stress in *A. annua* and a reduction of catabolic gene expression is adopted as a defense strategy in adverse conditions. Higher expression of biosynthetic genes and lower expression of catabolic genes at the preflowering stage confirmed the important role of proline in flower development. Artemisinin content decreased owing to water stress, but the slightly higher amounts were detected in leaves of severely stressed plants compared with moderately stressed plants. The artemisinin content of *A. annua* might be regulated by controlling irrigation regimes.

**Abbreviations**
*A. annua*: *Artemisia annua*
APX: ascorbate peroxidase
CAMAG: chromatographic twin‐trough vertical glass chamber
C: control
cDNA: complementary DNA
DHAR: dehydroascorbate reductase
DTT: dithiothreitol
DW: dry weight
EVS: early vegetative stage
FS: flowering stage
Glu: glutamine
GR: glutathione reductase
HPTLC: high‐performance thin layer chromatography
LVS: late vegetative stage
MC: melting curve
MDA: malondialdehyde
MDHAR: monodehydroascorbate reductase
MS: moderate water stress
Orn: orinithine
P5CDH: P5C dehydrogenase
P5C: proline‐5‐carboxylate
P5CR: 1‐pyrroline‐5‐carboxylase reductase
P5CS: 1‐pyrroline‐5‐carboxylase synthase
PDH: proline oxidase
PFS: preflowering stage
RT‐PCR: real‐time PCR
RWC: relative water content
SOD: superoxide dismutase
SS: severe water stress
TBARS: thiobarbituric acid reactive substances
δ‐OAT: ornithine‐δ‐aminotransferase

Drought is one of the most serious environmental stresses that adversely affect plant growth and development 1. Plants overcome water stress in part by producing secondary metabolites, which are compatible solutes, free‐radical scavengers, and transpiration reducers that protect cells from environmental stresses such as water deficit 2. Artemisinin is a notable secondary metabolite because it is an antimalarial drug isolated from *Artemisia annua* L., one of the top 10 medicinal crops of the New World 3. Although environmental stresses affect artemisinin production 4, a moderate level of a stress such as water deficit can actually increase the production of secondary metabolites in medicinal plants. Studies have indicated that abiotic stress induced accumulation of artemisinin in *A. annua*, but the effects of different levels of water stress on artemisinin content at different growth stages are still not understood 3.

Stressed plants produce compatible solutes, which can accumulate without disrupting intracellular biochemistry 6. Proline acts as an enzyme osmoregulator and cellular protectant under drought stress. Accumulation of proline during drought has been observed in several crops including *A. annua* 7. It is synthesized in the cytosol and probably in chloroplasts 8. Two pathways, ornithine (Orn) and glutamine (Glu), are used for proline biosynthesis in plants. The Orn pathway occurs in mitochondria and employs ornithine through the action of ornithine aminotransferase (OAT), which transaminates ornithine into pyrroline‐5‐carboxylate (P5C), which travels to the cytosol where it is changed to proline by 1‐pyrroline‐5‐carboxylase reductase (P5CR). As P5C is the mutual intermediate in both proline synthesis and catabolism, *OAT* is involved in both catabolism and anabolism of proline 10.

Under stress conditions, the Glu pathway is usually predominant, and glutamine is exploited as the main precursor of proline biosynthesis through the action of pyrroline‐5‐carboxylase synthase (*P5CS*) 11. The Glu pathway occurs in the cytosol and is controlled by two enzymes, *P5CS* and *P5CR* 12. *P5CS* is produced by two homologous genes and displays γ‐glutamyl kinase and glutamic‐γ‐semialdehyde dehydrogenase activities. Both *P5CS1* and *P5CS2* genes have diverse roles in plant abiotic stress responses 13. The *P5CR* enzyme is encoded by only one gene in most plant species that have been studied 9. While proline biosynthesis occurs in the cytosol, proline catabolism happens in mitochondria 14. The catabolic pathway of proline is controlled by the genes proline dehydrogenase (*PDH*) and proline 5‐carboxylate dehydrogenase (*P5CDH*). PDH oxidizes proline into proline‐5‐carboxylate 8. P5C is further changed to glutamate by the activity of *P5CDH* 15. Two genes encoding *PDH* (*PDH1* and *PDH2*) have been identified in *Arabidopsis*, whereas a single *P5CDH* gene has been recognized in *Arabidopsis* and tobacco 16. *PDH* transcription is reduced under water stress but increases during rehydration, thereby limiting proline shortages during abiotic stress 17.

Proline accumulation has been used to indicate drought resistance in a number of plants, although clear‐cut evidence showing the role of proline accumulation in stress adaptation has been questioned by some authors 18. An expression study of proline metabolic genes will increase our understanding of plant stress metabolic regulation and provide data for developing varieties with water stress tolerance so that this medicinal crop can better withstand drought conditions. This study was conducted to analyze expression of seven proline metabolic genes, including *P5CS1*,* P5CS2*,* P5CR*,* OAT*,* PDH1*,* PDH2*, and *P5CDH*. We provide the first report of water stress‐controlled expression of proline metabolic genes and artemisinin content in *A. annua*.

In the present study, drought stress and the associated change in artemisinin content was measured by using high‐performance thin layer chromatography (HPTLC), which is a routine analytical technique owing to its precision in quantifying small amounts of compounds 5. The effects of water stress on secondary metabolite production may indicate how careful water management could increase metabolite production.

## Materials and methods

### Plant material and growing conditions

*Artemisia annua* cv. CIM‐Arogya seeds were obtained from M/S Ipca Pvt. Ltd., Ratlam, Madhya Pradesh, India. The plants were grown in a greenhouse (Vista Biocell Pvt. Ltd., Noida, Uttar Pradesh, India) at Jamia Hamdard University in New Delhi, India.

Approximately, 12 seeds were sown in pots filled with lawn soil and manure (25 g·kg^−1^ of soil). Thirty days after sowing, plants were transplanted into separate pots. The plants were grown at ±27 °C, with relative humidity of approximately 70%, and photosynthetically active radiation of 220 μmol·m^−2^·s^−1^. Plants received 10.5 h of light per day.

### Water stress treatment

One month after transplantation, plants were exposed to water stress. Plants were classified into three groups on the basis of water stress treatments: control, moderately, and severely water‐stressed plants. Pot soil moisture was evaluated daily by calculating the soil water content percentage using following formula: Soil water content % = {(fresh soil weight‐dry soil weight)/fresh soil weight}*100. The soil moisture in moderately and severely water‐stressed pots was maintained at 55 ± 5% and 35 ± 5%, respectively. Pots were watered with 600–1200 mL of filtered water regularly (control, C), once a day (moderate water stress, MS), or once every two days (severe water stress, SS). Data were collected at four growth stages: early vegetative stage (EVS) at 4 months after sowing (MAS), late vegetative stage (LVS) at 6 MAS, preflowering stage (PFS) at 8 MAS, and flowering stage (FS) at 10 MAS. Data were collected from 12 plants for each of three treatments. Leaf samples from each treatment were collected, pooled, and used as one replication.

### Study of growth, leaf water status, and physiological parameters

#### Plant growth parameters

Plant growth parameters including height, distance between internodes, and lateral branch length were measured in cm with the ruler. Number of lateral branches was calculated numerically. Plant fresh weight (FW) was measured with a balance. Total plant dry weight (DW) (root + stem + leaf) reflected biomass accumulation. Leaf turgid weight was taken by placing leaf samples in 20 mL water into a Petri dish at 4 °C overnight. The following day, the samples were blotted dry and then reweighed to obtain turgid weight.

#### Leaf water potential, leaf area, and leaf water status

Leaf water potential was measured by Chardakov's method 19. Leaf mass fraction, relative water content (RWC), root mass fraction, shoot to root ratio, stem mass fraction, and leaf to stem ratio were calculated using the following formulas:

$$\text{Leaf mass fraction}=\text{Leaf dry mass/total plant dry mass}\;g\cdot g^{-1}$$

RWC=[(FW−DW)/(turgid weight−DW)]×100

$$\text{Root mass fraction}=\text{Root dry mass/total plant dry mass g}\cdot g^{-1}$$

$$\text{Shoot to root ratio}=\left(\text{Leaf}+\text{stem dry mass}\right)/\text{root dry mass}\;\;\;g\cdot g^{-1}$$

$$\text{Stem mass fraction}=\text{Stem dry mass}/\text{total plant dry mass}\;\;\;g\cdot g^{-1}$$

Leaf to stem ratio=dry mass of leaf/dry mass of stem

#### Physiological parameters

##### Chlorophyll estimation

The chlorophyll content was analyzed with Arnon's method 20. The amount of chlorophyll (chl a + b) was calculated and expressed as mg·g^−1^ FW.

##### Proline content

Proline content was estimated by the Bates *et al*. 21 method. Proline content was calculated by using the standard curve; 0, 10, 20, 30, 40, 50, and 60 μg of proline and recorded.

##### Total soluble proteins

Fresh leaves (0.5 g) were ground with 1 cm^3^ phosphate buffer (0.1 m, pH 7.0) in mortar and pestle and kept in ice. The concentration of proteins was determined by using a bovine serum albumin standard 22. Absorbance was read at 595 nm on a spectrophotometer.

##### Lipid peroxidation rate

Estimation of thiobarbituric acid reactive substances (TBARS) content followed the method of Cakmak and Horst 23. The obtained value was used for calculations with the following formula:

$$\mathrm{TBARS}\;\mathrm{content}(\mathrm{nmol}\cdot g^{-1}\mathrm{FW})=\left(A532-A600\right)V\times 1000/\varepsilon \times W$$

where, ε is the specific extinction coefficient (155 mm·cm^−1^), *V* is the volume of grinding medium, *W* is the FW of the leaf, A600 is the absorbance at 600 nm, and A532 is the absorbance at 532 nm.

##### Enzyme assays

An enzyme extract for the estimation of activities of antioxidative enzyme including superoxide dismutase (SOD), ascorbate peroxidase (APX), glutathione reductase (GR), monodehydroascorbate reductase (MDHAR), and dehydroascorbate reductase (DHAR) was made by freezing 1 g of leaf sample in liquid nitrogen and crushing the material in a phosphate extraction buffer. The extract was filtered, the filtrate was centrifuged at 15 000 ***g*** for 20 min, and the supernatant was used as the enzyme solution. SOD activity was assessed by observing the decrease in optical density of formazone formed by superoxide radicals and nitro blue tetrazolium dye 24. The absorbency was noted at 560 nm, and one unit of enzyme activity was considered the quantity of enzyme that decreased the absorbance by 50% in contrast to tubes lacking the enzyme solution. APX activity was analyzed using the method of Nakano and Asada 25 and recorded as the decline in optical density due to ascorbic acid at 290 nm 26.

Glutathione reductase was assayed using the method of Smith *et al*. 26. The increase in absorbance at 412 nm was recorded with a spectrophotometer. The activity was determined as total absorbance (∆*A*_412_) per milligram of protein per minute. MDHAR activity was determined by measuring the oxidation of reduced nicotinamide adenine dinucleotide following the method of Hossain *et al*. 27. The reaction was tracked by determining the decrease in absorbance at 340 nm. DHAR activity was determined by following the method given by Dalton *et al*. 28.

### RNA isolation and purification

Leaf tissue was collected from *A. annua* at different stages. Total ribonucleic acid (RNA) from 100 mg of tissue was extracted using RNeasy plant mini kit (Qiagen, Inc., Valencia, CA, USA) following the manufacturer's directions. The isolated RNA was quantified using a NanoDrop, and the integrity of the RNA samples was confirmed by visualizing the 28S and 18S rRNA bands on a 0.8% agarose gel after electrophoresis. These cDNA samples were used as templates in real‐time polymerase chain reaction (PCR) assays. The remaining samples of leaf tissues were oven dried and stored at 25 ± 3 °C in airtight boxes for use in HPTLC analysis.

### Reverse transcriptase‐mediated cDNA synthesis

Deoxyribonucleic acid (DNA)‐free total RNAs from treated and control plants were reverse transcribed into complementary DNA (cDNA) by reverse transcriptase. Reverse transcription of 50 ng (5 μL) of total RNA was conducted using 2 μL of random hexamer primer with the M‐MuLV RT‐PCR kit (Genei, Bangalore, India). The reagents were mixed and incubated at 65 °C for 10 min before the following reagents were added sequentially: 1 μL of RNAsin, 1 μL of 100 mm dithiothreitol, 4 μL of 5× reverse transcriptase buffer, 2 μL of 30 mm deoxyribose nucleoside triphosphate (dNTP) mix (7.5 mm of each dNTP), 1 μL of 100 U·μL^−1^ M‐MuLV reverse transcriptase, and 1 μL of nuclease‐free water. The reaction mixture was incubated at 37 °C for 1 h and then at 95 °C for 5 min. The tubes were then immediately cooled on ice for 15 min and stored at −20 °C until use.

### Primer design and real‐time PCR

All primers were designed using the PrimerQuest software tool and synthesized by IDT. The PCR products were designed to be ˂115 bp and primers were checked for specificity with BLAST. Primer sequences used in the study are provided in Table 1. For each primer pair, the consistency of the quantitative PCR (qPCR) was confirmed by amplification of the purified target sequence in a concentration series spanning six orders of scale. Linear regression analysis of the target concentration and the cycle threshold (Ct) value yielded correlation coefficients close to 1 for all primer pairs (Table 2), proving the efficiency of the PCR reaction.

**Table 1:** Forward (F) and reverse (R) primer sequences used for expression analysis

Gene	5′–3′ sequence
*P5CS1*	F‐GTAGACGACGACGACGATAATG
	R‐ACTGCTGTCCCAACCTTAAC
*P5CS2*	F‐GGTGCTGAGGTGGGAATAAG
	R‐ACTTGTCCCTTTCCTCTCATTATC
*P5CR*	F‐GAGGAGTAGCTGCTGGTTTAC
	R‐CCTGGATGCTTCCCAGTTT
*OAT*	F‐TGCTTGAGCTTGAAGGAGAG
	R‐GATCCATCTCGGAGTTCATCAG
*PDH1*	F‐AGCAGCTCATGGAAGGATTC
	R‐GGGTTGGAGGATTGTGTCTT
*PDH2*	F‐GCGTAGAACACGCTGAAGA
	R‐GCTTAAGTGAGACGAAGGTAGG
*P5CDH*	F‐AGGATGCACGCTCATCTAAC
	R‐CCAGCATATGTAGTCCCATTCA
*GAPDH*	F‐TTGTTGTTGAGTCCACTG
	R‐CTTGTATTCCTTCTCGTTGA

**Table 2:** Efficiency of the qPCR reaction for all primers used in the study

Gene	Slope	Amplification efficiency	% efficiency
*GAPDH*	−3.18	2.06	106.28
*P5CS1*	−3.19	2.06	105.82
*P5CS2*	−3.43	1.96	95.68
*P5CR*	−3.34	1.99	99.25
*PDH1*	−3.40	1.97	96.84
*PDH2*	−3.43	1.96	95.68
*OAT*	−3.38	1.98	97.63
*P5CDH*	−3.53	1.92	91.99

The expression of proline metabolic genes of different stressed leaf samples, as well as the control sample, was investigated using real‐time PCR (RT‐PCR; Light Cycler 480, Roche). The reaction mixture included 1 μL of each cDNA sample, 10 μL of SYBR green, 7 μL of water, and 1 μL of each specific forward primer. Cycle parameters were as follows: initial denaturation at 95 °C for 10 min, followed by 40 cycles of 95 °C for 15 s, 60 °C for 30 s, and extension at 72 °C for 30 s.

After amplification, melting curves were analyzed for all amplicons to confirm the precision of each reaction. The melting curve was obtained by heating the amplicon from 72 to 95 °C with a heating rate of 0.1 °C·s^−1^. The thermal profile of each reaction ended by cooling the amplicon to 40 °C for 10 s. The Ct values were the means of three independent PCRs for all seven genes and the endogenous control. Relative expression of genes among different leaf samples was calculated by the 2‐^ΔΔCT^ method of Livak and Schmittgen 29. The expression levels of the genes were normalized to the level of GAPDH and given on a logarithmic scale expressed as ∆∆Ct: $\Delta \Delta \text{Ct}=\left(\text{Ct of MS/SS}-\text{Ct of GAPDH}\right)-\left(\text{Ct of control}-\text{Ct of GAPDH}\right)$. The expression patterns of genes were measured on the basis of change in normalized ∆∆CT (≥ ±3) in the leaves of the control sample compared with leaves of MS and SS samples. Amplification proficiency of the PCR reaction was measured from the slope of the standard curves by the formula $E=10^{\left(-1/\mathrm{slope}\right)}$_,_ and *E* was converted to a percentage by $E=(10^{\left(-1/\text{slope})-1\right)}*100$, where slope was determined from the linear regression of target concentration (log) versus Ct.

### HPTLC analysis of artemisinin

#### Chemicals and artemisinin standard

All the chemicals used were of analytical grade and purchased from E. Merck. The HPTLC plates were layered with silica gel (G 60F254) and were purchased from E. Merck. The artemisinin standard was obtained from Sigma‐Aldrich (UK).

#### Sample preparation

Dried samples (100 mg) of *A. annua* were crushed with a mortar and pestle and sieved through a 1 mm mesh. The sieved powder was mixed with 10 mL acetone in a 30 mL glass vial. The mixture was microwaved at 190 W for 2 min. Each sample was syringe filtered into a test tube and 10 mL of acetone was added for an additional round of extraction. Samples were dried with a Rotavapor rotary evaporator. The residue was mixed in 2 mL methanol and filtered with a 0.22 μm syringe filter (Millipore, Merck, Billerica, MA, USA). The filtrate was stored at 4 °C until HPTLC analysis.

#### Standard solution preparation

One milligram of pure artemisinin was dissolved in methanol to obtain a volume of 1 mL and mixed by vortexing. This stock solution was diluted with methanol to prepare different working solutions, which were used as standards in the HPTLC densitometric study.

#### HPTLC analysis

High‐performance thin layer chromatography plates (20 cm × 10 cm) were activated in an oven at 110 °C for 30 min. Both sample and standard solutions were smeared on the plates in bands 6 mm wide and 10 mm apart using a CAMAG Linomat sample applicator equipped with a 100 μL syringe under a constant flow of N_2_ gas with a steady application rate of 150 μL·s^−1^. The bands were 10 mm apart, 8 mm from the bottom, and 10 mm from the left and right sides of the plate. The plate was developed in a normal chromatographic twin‐trough vertical glass chamber (CAMAG) that was pretreated for 30 min with 40 mL of mobile phase (9 : 1 toluene : ethyl acetate, v/v) at ambient temperature. Rising mode was used for plate development, with a distance of 90 mm. Following development, the HPTLC plate was air dried and immersed into freshly prepared anisaldehyde spraying mixture (50 : 1 : 0.5 glacial acetic acid:concentrated H_2_SO_4_:anisaldehyde, v/v/v) for postchromatographic derivatization followed by heating for 5 min at 110 °C to visualize the pink bands of artemisinin. Densitometric measurements were made at 536 nm using the tungsten lamp of a CAMAG TLC Scanner 3 in absorption‐reflection mode and wincats software, CAMAG, Muttenz, Switzerland (v.1.4.3.6335) at a wavelength of 536 nm. The slit dimension was 6.00 × 0.45 mm, with a scanning speed of 20 mm·s^−1^, and data step resolution of 100 μm. The plates were imaged with a CAMAG Reprostar 3 video camera.

#### Artemisinin calibration curve

Six volumes of standard working solution (1–6 μL) containing artemisinin (300, 600, 900, 1200, 1500, or 1800 ng per spot) were applied to HPTLC plates to make six points on a linear calibration curve for each volume of solution. The artemisinin calibration curve was determined by plotting peak area versus artemisinin concentration.

#### Quantification of artemisinin test samples

Each sample was applied twice in a 2 μL volume onto a HPTLC plate with the Linomat 5 applicator. The plate was developed and scanned as described in the section on HPTLC analysis, and peak areas were recorded. The artemisinin quantity was measured by comparison with calibration curves and was calculated as a percentage of DW.

#### Statistical analysis

All experiments were repeated three times, and data are presented as the mean and standard error of three replicates. Data were evaluated with a two‐way ANOVA using spss software (IBM, Armonk, NY, USA).

## Results

### Drought stress‐induced inhibition of plant growth

Water stress caused a significant (*P* < 0.05 level) reduction in plant height, branch number, lateral branch length, and internode length at all stages of development, and the reduction was more pronounced in severely water‐stressed plants. Biomass accumulation decreased with MS and SS in a stress severity‐dependent manner at all stages. The maximum reduction in biomass accumulation (79.09%) was observed under SS at LVS. Overall, water stress limited *A. annua* growth, and growth reduction was positively correlated with increasing water stress (Table 3).

**Table 3:** Effects of moderate and severe water stress on leaf water potential, total biomass accumulation, leaf to stem ratio, stem to root ratio, and relative water content in *A. annua* plants. EVS, early vegetative stage; LVS, late vegetative stage; PGS, plant growth stage; PFS, preflowering stage; FS, flowering stage; C, control; MS, moderate stress; SS, severe stress; T, treatment. (Values within parentheses are percent reduction (−) or increase (+) over control)

PGS	T	Leaf water potential (MPa)	Total biomass accumulation	Leaf to stem ratio	Stem to root ratio	Relative water content (MPa)
EVS	C	−0.32 ± 0.06	17.04 ± 1.52	0.31 ± 0.04	6.78 ± 2.89	43.20 ± 5.43
	MS	−0.40 ± 0.06 (−25.00)	11.2 ± 1.56 (−34.30)	0.35 ± 0.17 (+18.00)	6.94 ± 0.44 (−4.49)	40.90 ± 1.88 (−5.35)
	SS	−0.57 ± 0.06 (−75.00)	6.33 ± 0.43 (−62.85)	0.23 ± 0.09 (−25.91)	5.88 ± 1.59 (−17.15)	40.20 ± 3.94 (−7.03)
LVS	C	0.65 ± 0.06	93.30 ± 2.99	0.20 ± 0.03	12.70 ± 2.78	44.91 ± 3.43
	MS	−0.73 ± 0.10 (−13.00)	32.33 ± 2.73 (−65.30)	0.35 ± 0.02 (+75.00)	7.20 ± 1.70 (−43.10)	44.60 ± 5.97 (−0.66)
	SS	−0.81 ± 0.06 (−25.00)	19.51 ± 2.70 (−79.09)	0.36 ± 0.06 (+78.40)	8.38 ± 2.45 (−31.21)	45.75 ± 4.51 (+1.88)
PFS	C	−0.73 ± 0.10	160.88 ± 2.59	0.31 ± 0.03	8.30 ± 1.40	65.35 ± 4.94
	MS	−0.81 ± 0.06 (−11.10)	62.12 ± 2.56 (−61.40)	0.48 ± 0.03 (+52.00)	10.50 ± 2.52 (+28.30)	45.37 ± 1.75 (−30.60)
	SS	−0.89 ± 0.06 (−22.20)	58.10 ± 2.59 (−63.90)	0.40 ± 0.09 (+26.90)	12.51 ± 3.51 (+53.70)	43.40 ± 3.35 (−33.60)
FS	C	−1.05 ± 0.06	68.93 ± 2.99	0.31 ± 0.02	12.90 ± 2.82	70.06 ± 1.28
	MS	−1.13 ± 0.06 (−7.70)	29.98 ± 2.67 (−56.50)	0.37 ± 0.09 (+23.00)	6.67 ± 2.07 (−47.90)	55.59 ± 3.37 (−20.70)
	SS	−1.21 ± 0.10 (−15.4)	17.40 ± 2.13 (−74.76)	0.38 ± 0.09 (+23.50)	6.73 ± 1.08 (−48.60)	52.59 ± 1.98 (−24.93)

### Effect of water stress on leaf water status

Leaf water potential and relative water content were lowered by water stress (Table 3). Maximum leaf water potential was lowered by 75% under SS at EVS, whereas the least reduction (7.7%) was observed under MS at FS. The RWC displayed a similar pattern and was reduced under MS and SS, with a greater reduction in SS plants except at LVS, when RWC was 1.88% higher than in the control (Table 3).

### Effects of water stress on chlorophyll, proline, total soluble protein, and TBARS

**Figure 1:**
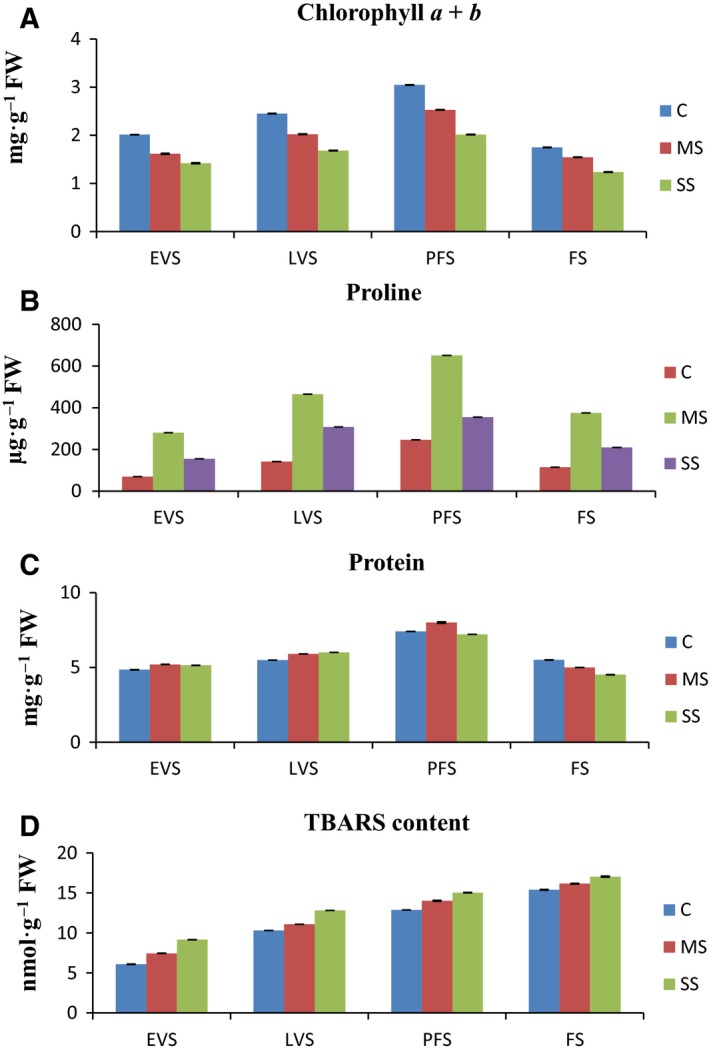
(A–D) Effects of water stress on chlorophyll, proline, total protein, and thiobarbituric acid reactive substances (TBARS) content at different development stages of *A. annua*. EVS, early vegetative stage; FW, fresh weight; LVS, late vegetative stage; PGS, plant growth stage; PFS, preflowering stage; FS, flowering stage; C, control; MS, moderate stress; SS, severe stress.

Total chlorophyll (Chl a + Chl b) content in the leaf samples was reduced in both MS and SS samples. The maximum reduction (1.24 mg·g^−1^ FW) was observed under SS at FS, whereas the minimum reduction (2.53 mg·g^−1^ FW) was observed under MS at PFS (Fig. 1A). Proline content was enhanced in MS and SS plants. The maximum proline level (650.07 μg·g^−1^ FW) was observed under MS at PFS (Fig. 1B). Total soluble protein content was higher under MS followed by C and SS at PFS, whereas it decreased at FS in both MS and SS plants (Fig. 1C). TBARS content was likewise enhanced at all developmental stages in MS and SS plants. The maximum increase in TBARS content was found under all stages at FS, whereas the minimum increase was observed under C and MS at EVS (Fig. 1D).

### Effect of water stress on antioxidant activity

**Figure 2:**
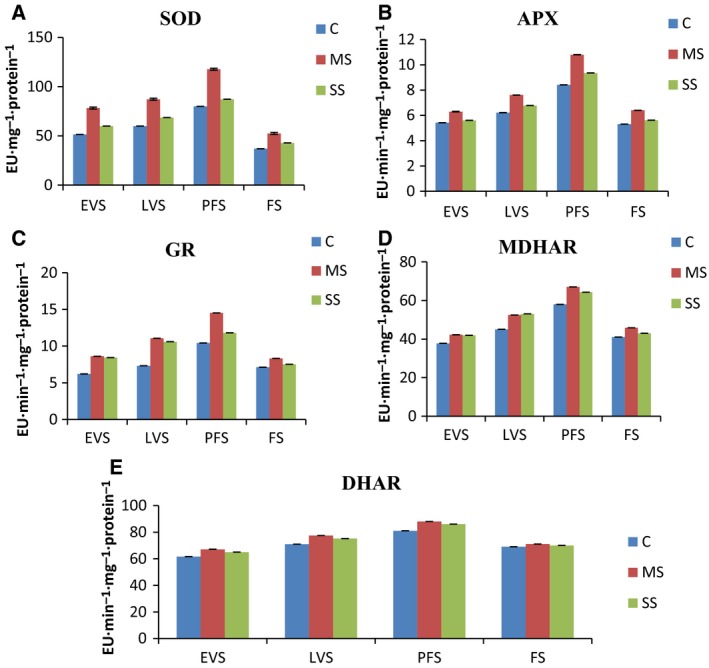
(A–E) Effects of drought stress on activities (Enzyme Units, EU) of superoxide dismutase, SOD (A), ascorbate peroxidase, APX, (B) glutathione reductase, GR, (C), monodehydroascorbate reductase, MDHAR (D), and dehydroascorbate reductase, DHAR (E) in leaves of *A. annua*. EVS, early vegetative stage; FW, fresh weight; LVS, late vegetative stage; PGS, plant growth stage; PFS, preflowering stage; FS, flowering stage; C, control; MS, moderate stress; SS, severe stress.

Drought‐stressed plants showed increased SOD activity, but the increase was less under SS than MS at all stages. Maximum increase (117 mg^−1^·protein^−1^) was observed under MS at PFS (Fig. 2A). A similar pattern was observed for APX activity, with an increase in enzyme activity that was highest in MS plants at PFS (Fig. 2B). Both MS and SS plants showed increased GR activity, with the highest increases at PFS (Fig. 2C). MDHAR and DHAR activity increased for all water stress treatments at all stages (Fig. 2C,D).

### Expression of proline biosynthesis and catabolic genes in response to water stress

**Figure 3:**
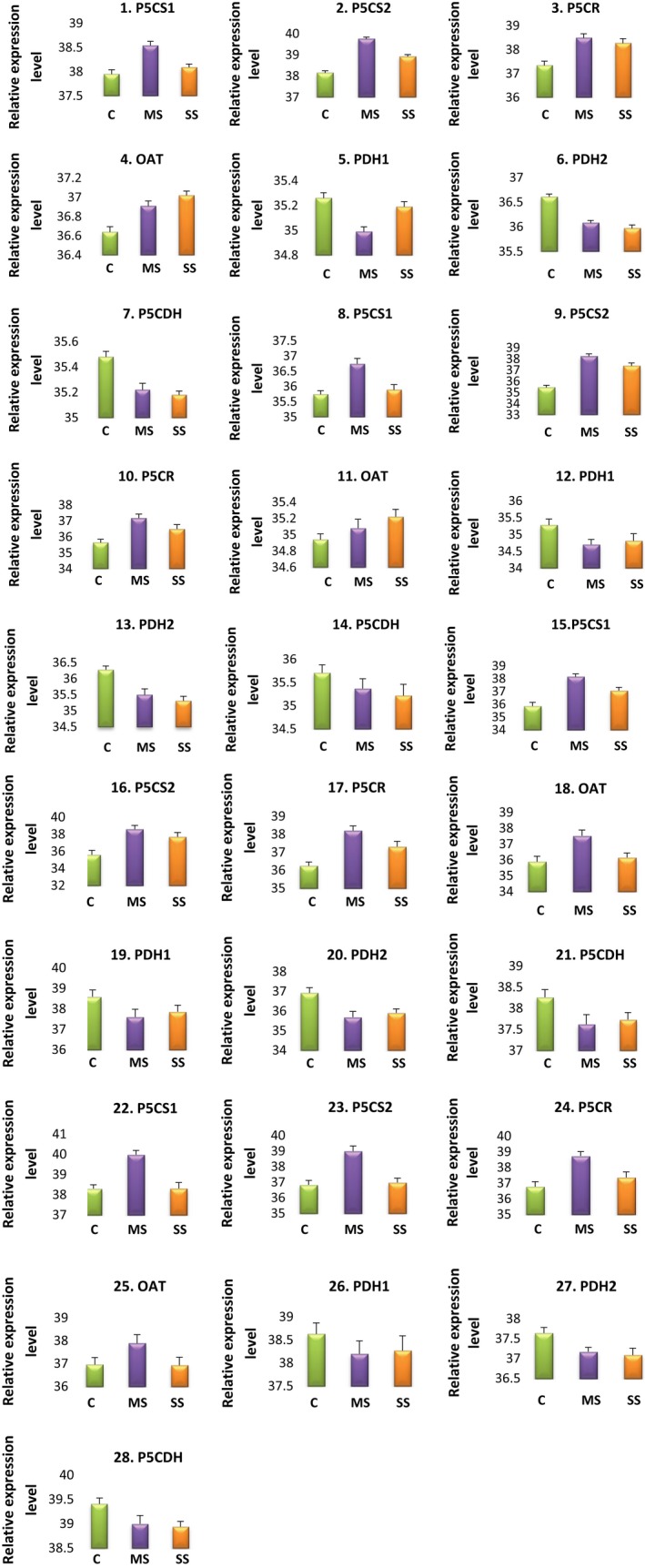
Relative quantification plot of proline metabolic genes among the control (C), moderately stressed (MS), and severely stressed (SS) *A. annua* plants at different developmental stages. 1–7, early vegetative stage; 8–14, late vegetative stage; 15–21, preflowering stage; 22–28, flowering stage. The expression levels of mRNAs were normalized to the level of GAPDH and given on a logarithmic scale expressed as 45‐∆CT, where ∆CT is the difference in real‐time PCR threshold cycle number of the respective gene and the reference gene, where 45 equals the expression level of GAPDH gene (the number 45 was chosen because the PCR run stops after 45 cycles). The results are averages ± SE of duplicates of three samples.

Relative quantification plots of proline metabolic genes in the control and MS and SS treatments are shown in Fig. 3. The RT‐PCR results indicated that proline biosynthetic genes, including *P5CS1*,* P5CS2*, and *P5CR*, were induced under water stress at all developmental stages, while *OAT* was reduced 0.98‐fold under SS at FS (Table 4). The transcript level of *P5CS1* increased greatly (*P* < 0.05 level) at all stages, although the increase was greater under MS than under SS. A maximum increase of 4.93‐fold was observed under MS at PFS, whereas no change was observed at FS in SS plants. Similarly, the expression level of *P5CS2* under water stress was increased 1.1‐ to 8‐fold under SS at FS and MS at PFS, respectively. Likewise, a 3.89‐fold upregulation of *P5CR* was observed under MS at PFS.

**Table 4:** Differentially expressed proline metabolic genes and their expression levels in water‐stressed and control leaf samples of *A. annua*. EVS, early vegetative stage; LVS, late vegetative stage; PGS, plant growth stage; PFS, preflowering stage; FS, flowering stage; C, control; MS, moderate stress; SS, severe stress

Gene	Fold increase (+)/decrease (−) in MS and SS compared to control							
	EVS		LVS		PFS		FS	
	MS	SS	MS	SS	MS	SS	MS	SS
*P5CS1*	(+1.51)	(+1.10)	(+2.00)	(+1.11)	(+4.92)	(+2.31)	(+3.20)	a
*P5CS2*	(+3.03)	(+1.71)	(+6.92)	(+3.81)	(+8.00)	(+4.32)	(+4.50)	(+1.11)
*P5CR*	(+2.22)	(+1.91)	(+2.91)	(+1.80)	(+3.89)	(+2.09)	(+3.9)	(+1.51)
*OAT*	(+1.21)	(+1.30)	(+1.10)	(+1.21)	(+3.09)	(+1.19)	(+1.91)	(−0.98)
*PDH1*	(−0.83)	(−0.95)	(−0.67)	(−0.72)	(−0.50)	(−0.60)	(−0.74)	(−0.78)
*PDH2*	(−0.69)	(−0.64)	(−0.58)	(−0.51)	(−0.42)	(−0.49)	(−0.72)	(−0.68)
*P5CDH*	(−0.84)	(−0.81)	(−0.79)	(−0.71)	(−0.64)	(−0.69)	(−0.75)	(−0.72)

a No change as compared to control.

In contrast, the expression patterns of the proline catabolic genes *PDH1*,* PDH2*, and *P5CDH* were downregulated under water stress conditions (Table 4). Leaves of SS plants showed the highest reduction in expression of proline catabolic genes. *PDH* gene expression varied at different developmental stages under water stress condition. The highest reduction (0.95‐fold) in *PDH1* transcript was found at EVS under SS, whereas the lowest reduction (0.50‐fold) was observed at PFS under MS. *PDH2* showed a similar trend at PFS under MS, but the maximum reduction (0.72‐fold) was observed at FS under MS. The expression pattern of *P5CDH* was similar, with a 0.84‐fold reduction at EVS under MS and a minimum reduction (0.64‐fold) at PFS under MS. The changes in the transcript levels of *PDH1, PDH2*, and *P5CDH* are shown in Fig. 3.

### HPTLC analysis of water stress‐influenced artemisinin content

**Figure 4:**
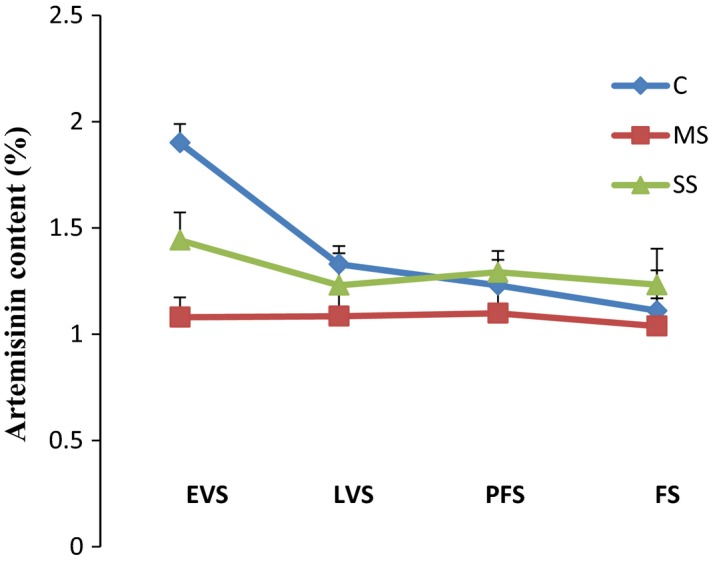
Artemisinin content in control (c), moderately stressed (ms), and severely stressed (ss) *A. annua* plants. EVS, early vegetative stage; FW, fresh weight; LVS, late vegetative stage; PGS, plant growth stage; PFS, preflowering stage; FS, flowering stage; C, control; MS, moderate stress; SS, severe stress. The values are means of three replicates.

Detailed TLC studies revealed that bands of artemisinin were well separated on the HPTLC plate, with a retention factor of 0.27 ± 0.03. Fingerprint patterns of the test samples obtained under identical conditions showed that the amount of artemisinin varied at the different developmental stages (Fig. 4). The artemisinin percentage was lowered by moderate water stress conditions. Artemisinin content expressed as the percentage per g DW was initially increased at moderate stress level with plant developmental stages including EVS, LVS, PFS but in later FS stage, it found decreased (Fig. 4). However, the artemisinin percentage was significantly lowered under water stress conditions, although values were higher under SS than MS at all stages. Artemisinin content was highest (1.9%) at EVS for the control plants, whereas minimum content (1.038%) was detected at FS under MS (Fig. 4).

## Discussion

### Effect of drought stress on overall plant growth

The primary effect of dehydration is a reduction in cell turgor, which reduces overall growth 30. Water deficit negatively affected plant growth including height, branch number, internode length, and lateral branch length. Plants can survive under extreme conditions in part by growing more slowly. Growth reduction was higher in plants grown in 35 ± 5% soil water content than in 55 ± 5%. Severe water stress reduces water use to such a level that the drought tolerance mechanisms are inadequate to sustain ordinary growth. A decrease in growth in water‐stressed plants is attributable to the low turgor pressure that results from low soil water availability 31, and water‐stressed plants tend to be smaller. Medeiros *et al*. 32 have confirmed a drought stress‐related decline in the growth of a diversity of plants. Water stress commonly reduces the FW of crop plants 33. In the present study, reductions in root and shoot FW were recorded in both moderately and severely stressed conditions. Dehydration causes a noteworthy reduction in total dry biomass and halts the overall growth of the plant 34. Total biomass reduction was less in MS plants than in SS plants except at PFS, where the difference between treatments was not significant (*P* < 0.05 level). The stress response is determined by the developmental stage of the plant. A similar response was found in wheat 35.

### Effects of water stress on leaf water status

Low moisture increases the leaf vapor pressure, which may increase leaf transpiration and conductance, reducing leaf water potential. Leaf water potential (Ψ_w_) was higher in well‐watered plants than in the stressed plants. The severe water deficit, with 35 ± 5% soil moisture caused a smaller reduction in water potential than the moderate water deficit. Marchese *et al*. 36 observed leaf Ψw −1.39 and −2.51 MPa, respectively after introducing water deficit treatments of 38 and 62 h in *A. annua*. In the current study, a maximum Ψ_w_ of −1.2 MPa was recorded under SS plants at FS. Control plants at all stages had low Ψ_w_ values. These results are in agreement with those from Barbados cherry 32 and *A. annua* 36. Leaf RWC was lower after water stress, and the lowest value was found under SS at PFS; this outcome shows that a reduction of RWC is connected with the severity of stress. These results agree with those of Lobato and Costa 37, who found a reduction in leaf RWC due to water stress in cowpea. Similar results were obtained in common bean 38.

### Effects of water stress on chlorophyll, proline, total soluble protein, and TBARS

Chlorophyll content reflects a plant's physiological status 39. Under water stress, there was a reduction of Chl a + b under MS and SS, but the percentage reduction was significantly higher under SS at all developmental stages (Table 3; *P* ≤ 0.05). Al‐Absi 40 documented a similar decline in growth of oranges under severe water stress, linked with a reduction in chlorophyll content.

Plants produce compatible solutes during water stress to sustain cell turgor when water potential is low 18. Proline accumulation during water stress has been documented in many species and has an important role in osmotic change 39. In this study, osmotic potential was reduced as free proline increased significantly (*P* < 0.05 level) in the MS and SS plants. In agreement with our findings, Nogueira *et al*. 41 recorded a 38.1% increase in proline in stressed Barbados cherry compared to the control. Similarly Yadav *et al*. 42 observed increase in proline content under water stressed plants. Total soluble protein content increased at earlier stages (EVS and LVS) under MS and SS, but it began to decrease at PFS under SS and continued to decrease until FS in both treatments. The early increase in total soluble proteins during drought may be due to the expression of new stress proteins, but the reduction was due to a severe decrease in chlorophyll content, which reduced photosynthesis and altered carbon and nitrogen metabolism. The increase and reduction in total soluble proteins under drought was comparable with results from maize 43. Drought may interrupt regular reactive oxygen species (ROS) equilibrium and stimulate lipid peroxidation of membranes, either by increased production or reduced scavenging of ROS in the cell. The level of malondialdehyde (MDA) in plant tissues is considered the physiological marker of lipid peroxidation 44. In the current study, drought stress increased MDA levels, indicating membrane injury; the increase was more pronounced under SS than MS. Tatar and Gevrek 45 also reported that MDA content in wheat leaves increased with drought stress severity.

### Effects of water stress on antioxidant activity

It is widely acknowledged that ROS cause lipid peroxidation and stress‐induced damage to macromolecules 46. Thus, the function of antioxidative enzymes, such as SOD, GR, APX, MDHAR, and DHAR, is vital. These enzymes run the ascorbate–glutathione cycle or Foyer–Halliwell–Asada pathway 47. Drought stress can increase the production of ascorbate–glutathione cycle enzymes in various plants 48. SOD is the principal enzyme in removing ROS and converts O_2_^−^ to H_2_O_2_ in the cytosol, chloroplast, and mitochondria, and plays a critical role in cellular defense mechanisms against OH^−^ formation 49. Improved SOD activity in *Brassica oleracea* L. 50 was noted under drought treatments. In our research, water stress significantly (*P* < 0.05 level) increased SOD activity in MS and SS plants. APX is actively involved in the removal of ROS in the chloroplast and cytosol, breaking down H_2_O_2_ to form H_2_O and monodehydroascorbate 51; therefore, this enzyme has an important role in tolerance to drought conditions. In the current study, APX increased in plants under drought stress conditions. Yang *et al*. 52 noted an increase in APX activity under low water conditions in *Phaseolus vulgaris*. The relatively low activity of APX at FS, compared to other stages, might reflect the low antioxidant levels normally found in mature leaves, which makes them susceptible to greater oxidative damage than young leaves. Similar results were observed by Oberoi *et al*. 53 in chickpea, and the authors discussed the importance of upregulation of diverse antioxidant enzymes at different stages of leaf development. GR is a flavor‐protein oxidoreductase that acts as a crucial component of the cellular defense system against ROS by sustaining protective antioxidants like ascorbate and glutathione, which detoxify the damaging ROS. Typically, GR functions in a cycle with APX. In the present investigation, the activity of GR increased in leaves of *A. annua* under water stress conditions and was higher at EVS and LVS than in later stages of development.

Oberoi *et al*. 53 also studied stage‐specific upregulation of the antioxidant defense system, including GR, in chickpea leaves. The increase in GR activity might be linked with more efficient synthesis of GR during drought stress. Thus, higher GR activity may stop the development of free radicals in the plant under drought stress conditions. MDHAR and DHAR are vital enzymes of the ascorbate–glutathione cycle that contribute to H_2_O_2_‐scavenging pathways in plant cells. In the current study, drought significantly increased MDHAR and DHAR activity in *A. annua*. Eltayeb *et al*. 54 reported that transgenic tobacco that overexpressed MDHAR had improved osmotic stress tolerance.

### Effects of water stress on proline metabolic genes

We found that the proline biosynthesis genes *P5CS1* and *P5CS2* were upregulated up to 4.92‐fold and 1.1‐ to 8‐fold, respectively, in water‐stressed plants, but induction of *P5CS2* was higher than *P5CS1*. The induction of *P5CS1* and *P5CS2* continued until PFS under MS and SS, whereas at FS, both genes showed comparatively low expression. The enzyme *P5CS* is rate‐limiting in proline synthesis. In many plant species, *P5CS* is encoded by two genes that have different expression patterns, and the enzymes have nonredundant roles of decreasing stress damage to the cell during development 55. Drought‐induced upregulation of *P5CS1* and *P5CS2* was detected in many plants including *Kosteletzkya virginica* (L.) C. Presl ex A. Gray 56 and tobacco 57.

In water‐stressed leaves of *A. annua*,* P5CR* transcript levels were increased following stress treatment, but the comparative expression level was less under SS at all developmental stages. The greatest increase (3.89‐fold) was recorded under MS at PFS and FS, followed by a 2.98‐fold increase under MS plants at LVS (Fig. 3). The transcript levels of *P5CR* increased with osmotic stress in prairie Junegrass 8 and *Arabidopsis* 55. De Ronde *et al*. 58 overexpressed *Arabidopsis P5CR* in soybean, and the transgenic plant showed improved drought tolerance, indicating that expression of antisense *P5CR* leads to stress sensitivity. Increases in proline during water deficiency form part of the metabolic defense system against drought stress.

Several studies have verified that drought stress increases δ*‐OAT* expression 59, 60. Upregulation of δ*‐OAT* expression may contribute to the accumulation of proline in response to drought stress 11. In the present study, *OAT* showed differential transcript level regulation. It was induced by water deficit at all stages except FS, where the transcript level was slightly downregulated (0.95‐ and 0.98‐fold under MS and SS, respectively). However, upregulation of *OAT* was less than that of other proline biosynthetic genes, indicating that the Orn pathway was subsidiary for proline accumulation in leaves under drought conditions. This is consistent with other findings, which suggested that the Glu pathway is the most important under drought stress 12. In contrast, catabolic pathway genes including *P5CDH*,* PDH1*, and *PDH2* were downregulated under drought in *A. annua*. Fig. 3 shows variations in the expression pattern of *P5CDH, PDH1,* and *PDH2*. *PDH*, which is encoded by two genes, plays a key role in proline catabolism 17. Our results demonstrated that water stress decreased both *PDH1* and *PDH2* transcript level at all stages, but the downregulation was greater for *PDH1* (0.50‐fold) than for *PDH2* (0.42‐fold). It is not clear how these genes are regulated by water stress, but the reduction of *PDH* expression during abiotic stress has been reported in different crops 12, 61. Rayapati and Stewart 62 hypothesized that the decrease in *PDH* activity might have been caused by a specific change in the mitochondrial inner membrane.

Regulation of *P5CDH* under drought stress varies in different crops. Sharma and Verslues 59 reported upregulation of *P5CDH* under low water potential, whereas some reports have indicated that *P5CDH* expression is repressed by osmotic stress 14. Our results indicated that this catabolic enzyme was downregulated 0.64‐ to 0.83‐fold under drought stress at different growth stages (Fig. 3). To support proline accumulation under adverse conditions, the catabolic enzymes P5CDH and *PDH*, situated in the mitochondria, are suppressed while the elevated level of proline contributes to redox buffering and other metabolic roles; this is likely an adaptive response to stress 59. Moreover, our data indicate that the dynamics and expression levels can vary greatly depending on the drought stress severity and the developmental stage of the plant. High expression of proline biosynthetic genes and repression of proline catabolic genes in water‐stressed plants at PFS suggested an important role of proline during this stage.

### Effect of water stress on artemisinin content

In our experiment, water stress decreased the artemisinin content in treated plants compared to control plants at all growth stages (Fig. 4). Though artemisinin was adversely affected by both MS and SS, there was a slight increase (11–23%) in artemisinin content in leaves under SS compared with MS plants. The reduction in artemisinin under MS was higher in earlier growth stages of the plants (EVS > LVS > PFS > FS).

The slight increase in the leaf artemisinin content as a result of severe water stress might reflect reduced growth under water stress. Excess photoassimilates would be incorporated into secondary metabolites 3, such as artemisinin. Munné‐Bosch *et al*. 63 found similar results in a drought‐resistant shrub, *Cistus creticus* L. Plants under drought stress usually accumulate abscisic acid, which activates changes in the content of secondary metabolites 64. However, different studies have shown inconsistent artemisinin contents under water stress conditions. Marchese *et al*. 36 stated that water stress for a short duration enhanced artemisinin content, whereas Yadav *et al*. 42 postulated that prolonged water stress negatively affected artemisinin content. Apparently, drought effects on artemisinin content vary depending on the time of harvest, light intensity, climate, developmental stage, and drought severity. This study emphasizes that drought reactions in secondary metabolism are very complex, and it may be possible to manipulate the artemisinin content of *A. annua* by limiting irrigation.

## Conclusions

The effects of water stress on a number of growth and physiological parameters and antioxidants were considered in *Artemisia annua*. Drought stress resulted in overall growth reduction, increased lipid peroxidation, and decreased chlorophyll content. Proline, soluble proteins, and antioxidant levels increased significantly in response to water stress. This study supports the theory that proline has a vital role in osmoprotection in *A. annua*. Proline accumulated in leaves, especially during inflorescence development, and is likely a defensive adaptation to avoid leaf tissue damage from drought. Under moderate stress, biosynthetic genes were highly expressed, while expression of catabolic genes was relatively low. Proline accumulated after proline synthesis via the Glu and Orn pathways, but the Glu pathway was more predominant.

Further investigation is needed to identify signaling components for regulating proline gene expression under water stress as well as during stress recovery in *A. annua*. An efficient genetic transformation protocol to transform *A. annua* plants with transgenes encoding the enzymes for the rate‐limiting steps of the proline metabolic pathway might lead to increased activity of proline and drought tolerance in *A. annua in vivo*. Artemisinin content was reduced under water stress conditions, but a slight enhancement was observed in severely stressed plants. The artemisinin content of *A. annua* might therefore be regulated through controlled irrigation.

## Author contributions

PS carried out laboratory work and drafted the manuscript. MZA conceived the idea and designed the experiment. All authors read and approved the final manuscript.
